# Personality and teachers’ burnout stress: exploring the digital competence as personal job resource in allied health institutions

**DOI:** 10.3389/fpsyg.2024.1334371

**Published:** 2024-05-09

**Authors:** Zawar Hussain, Cai Chenmei, Muhammad Saeed, Nazia Hassan, Fiza Chiragh

**Affiliations:** ^1^School of Education, Guangzhou University, Guangzhou, China; ^2^School of Management, Harbin Institute of Technology, Harbin, China; ^3^Department of Education, Allama Iqbal Open University, Islamabad, Pakistan

**Keywords:** big-five personality traits, burnout, digital competence, allied health, teachers’ stress, job demands and resources theory

## Abstract

**Introduction:**

Job burnout has severe consequences for teachers and students. This study aimed to measure the direct effects of personality traits on job burnout-stress syndrome among allied health educators. Furthermore, teachers’ digital competence was evaluated as a personal job resource for mitigating the negative impact of burnout.

**Methods:**

This study examined direct relationships between work-related stress syndrome and personality traits, namely, extroversion, agreeableness, conscientiousness, emotional stability, and openness to experience. Data was collected from 334 allied health institution teachers through a self-reported questionnaire. Linear regression analysis was used to test for direct effects. Moderating effects were evaluated using Andrew F Hays PROCESS macro v2.16.3.

**Results:**

All five personality traits had a significant negative relationship to burnout and teachers’ digital competence moderated the relationship between personality traits and burnout. This study’s findings provide evidence, that personality is significantly related to job burnout among allied health educators.

**Conclusion:**

These empirical findings conclude that personality traits are related to burnout in the non-Western culture of Pakistan. Furthermore, teachers’ digital competence acts as a personal job resource and potential moderator in the current digital working environment. Therefore, future teachers should enrich their digital competencies for improved performance, and advanced digital competency courses should be included in their curriculum.

## 1 Introduction

Working as a teacher is a highly psychologically demanding job, resulting in increased burnout stress (Fernet et al., 2012; Redín and Erro-Garcés, 2020). Burnout manifestations have been reported in approximately 30–40% of the teacher population, with severe consequences (García-Carmona et al., 2019). Teachers working in allied health institutions are no exception to severe burnout symptoms. The healthcare system is among the most rapidly changing professions in the world with critical importance (Torkaman et al., 2017). Therefore, teaching in allied healthcare institutions is a specialized and challenging job, susceptible to burnout (Torkaman et al., 2017; Chao et al., 2023). Teachers’ burnout leads to undesirable outcomes such as low motivation, loss of job performance, increased sickness rate, and perceived low professional efficacy (Swider and Zimmerman, 2010; Maslach and Leiter, 2016). This, in turn, affects the quality of teaching in allied health settings (Chao et al., 2023). Students are also experiencing these symptoms, with changes in teacher performance correlating with decreased student motivation and academic performance (Klusmann et al., 2016, 2021).

The World Health Organization (WHO) defines burnout as an occupational phenomenon and a chronic workplace stress syndrome that remains inadequately managed (World Health Organization, 2019). This condition is characterized by three primary symptoms: emotional exhaustion, depersonalization or cynicism, and low personal accomplishment (Maslach et al., 2001). Emotional exhaustion is a key initial symptom of burnout, characterized by a sense of emotional overload and lack of energy. Depersonalization involves distancing oneself and fostering a negative attitude toward colleagues. These include disengagement, lack of empathy, and negative perceptions of others. Lastly, low personal accomplishment manifests as feelings or perceptions of inefficiency and a diminished belief in one’s capacity to fulfill organizational responsibilities. Therefore, evaluating the factors related to burnout is crucial for researchers, serving as a foundational step toward intervention and mitigating the adverse effects of burnout on both teachers and students.

Personality is an important factor in shaping workplace behavior. Theoretical models emphasize individual differences as important factors in determining reactions to job-related stress. According to Lazarus’s transaction model of stress and coping (Lazarus and Folkman, 1984), experiencing stress results from an individual’s evaluation of environmental job demands and personal resources, which are inherently influenced by personality traits. Personality is a “unique psychological quality that influences an individual’s behavior, thoughts, and feelings through situations and times” (Roberts and Jackson, 2008). Among the prevailing personality models, five personality traits are considered dominant and comprehensive (John et al., 1991, 2008). A trait is defined as an observable behavior used to describe an individual (Lewis et al., 2001). The Big Five personality model is characterized by five personality traits: extroversion, agreeableness, conscientiousness, emotional stability, and openness to experience (McCrae and Costa, 2003).

Theoretical models link job stressors such as work overload, work-life conflict, role ambiguity, and low job control as determinants of burnout, often giving less emphasis to individual factors (Maslach et al., 2001; van Zoonen et al., 2021). However, the theoretical model of stress development reports that individuals respond differently to work stressors based on individual differences, such as personality traits (Lazarus and Folkman, 1984; Lazarus, 2006; Pérez-Rodríguez et al., 2019). Therefore, this study examined the contradictory viewpoints of theoretical models by examining the direct relationship between the Big Five personality traits and teacher burnout. Furthermore, exploring the relationship between personality traits and burnout stress remains an area of interest in recent times. Many studies examined the relationship between personality traits and burnout stress in different work groups (Dionigi, 2020; Bhowmick and Mulla, 2021; De la Fuente-Solana et al., 2021). Especially, studies explored the relationship between personality traits and burnout stress in professional education and medical practitioners to understand this complex relationship (Narang et al., 2022; Wang et al., 2022). However, recent studies encouraged more work to examine this relationship for mitigating burnout symptoms in the workplace (Roloff et al., 2022; Barends et al., 2024). This study examined the moderating role of digital competence as a personal job resource. According to the Job Demands-Resources (JD-R) model theory, job resources have two interactional effects: boosting and buffering. These attributes of job resources potentially mitigate the negative impact of burnout (Bakker and Demerouti, 2013; Tadić et al., 2015). Personal job resources, including digital competence, may assist employees in preventing the adverse effects of fatigue and persistent burnout (Bakker and de Vries, 2021). Teachers’ digital competence involves the use of digital technologies for implementing information and communication technologies (ICTs) and other digital tools for teaching and learning purposes (Knutsson et al., 2012). Educators and learners interact, collaborate, and use technological resources to address challenges. In this digital age, it is common for teachers and students to use digital tools for various learning tasks (Viberg et al., 2020). Thus, mastering the use of digital learning tools has become an essential competency for teachers (Julien and Barker, 2009). Therefore, in this study, teachers’ digital competence was evaluated as a personal job resource, following the proposal by Juyumaya and Torres (2020) regarding its status as a new job resource. Finally, the literature related to personality trait theory and job burnout originates extensively from the Western, Educated, Industrialized, Rich, and Democratic (WEIRD) countries. The applicability and generalization of these findings in non-Western culture is questionable (Henrich et al., 2010). Teaching practices, student classroom behaviors, educational institutional culture, and contexts, especially within allied health teaching institutions, often differ among countries (Klassen et al., 2018). Consequently, there is a need for research on teacher personalities and burnout in countries with diverse socioeconomic statuses (Kim et al., 2019).

Previous studies have primarily focused on organizational and environmental factors as determinants of teacher burnout often neglecting individual factors such as personality as a potential factor in the development of teachers’ burnout (Roloff et al., 2022). In literature, the role of the work environment factors as predictors of this syndrome is well-established (Lee and Ashforth, 1996; Maslach et al., 2001). However, the impact of personality on the development of burnout cannot be overlooked. Even when stressors in the work environment are reduced or eliminated, certain individuals may still experience elevated levels of burnout (Alarcon et al., 2009). This underscores the complex role of individual personality traits (extroversion, agreeableness, conscientiousness, emotional stability, and openness to experience) in the manifestation of burnout (Angelini, 2023b) and needs more consideration for better understanding of the relationship between personality and burnout (Barends et al., 2024). Therefore, the present study aims to examine the inter-individual factor of personality traits as an antecedent of teachers’ burnout in allied health institutions. This focus is particularly relevant in the non-Western culture of Pakistan, with an entirely different socioeconomic perspective from that of WEIRD countries. Additionally, this study examines the potential buffering role of digital competence as a personal job resource. Before this research, digital competence had not been assessed as a job-related resource to mitigate the effects of job burnout in teachers of allied health institutions.

This article makes several noteworthy scientific contributions. First, it adds to the existing literature on the correlation between personality traits and burnout. Second, this study extends the job demands and resources theory by examining digital competence as a personal job resource in allied health institutions. Thirdly, the study includes robust statistical analysis and empirical evidence, shedding light on the effects of personality traits on burnout in emerging nations, like Pakistan.

## 2 Literature review

### 2.1 Personality and burnout

Personality theories suggest that individuals’ dispositions affect how they interpret and react to their environment. Mischel and Shoda (1998), theorized in their cognitive-affective personality system (CAPS) that individual personalities influence how individuals encode and evaluate information received from the environment. They argue that an individual’s fear, frustrations, and impulsive behaviors are controlled by the mental encodings of their beliefs. These encodings are psychological reactions toward events and self-regulation patterns (Mischel and Ayduk, 2002). While it has never been reported, burnout symptoms may be considered as a set of individual mental encodings that they react to when experiencing workplace stress. Examining how people manage and react to stress, Mischel and Shoda (1998) discuss the value added by dispositional and process-based investigations. Job burnout may support dispositional- and process-based models for understanding an individual’s behavior at work in terms of coping. Consequently, we aim to describe how an individual’s stable personality traits affect their reactions to situational elements at work and their ensuing behavior. For instance, less emotionally stable individuals may interpret and encode changes in their work environments differently when compared to more emotionally stable people, who experience the same changes and respond differently to the same work environment. Thus, we assumed that personality traits would be related to job burnout (Swider and Zimmerman, 2010).

In exploring personality, the five-factor model is mostly accepted and famous approach commonly cited as the Big-Five personality traits model (Angelini, 2023a). The reason behind the acceptance of the five-factor model is its reliability across different cultures and stability over the years (Costa and McCrae, 1988; Mccrae and Costa, 1997; Angelini, 2023b). This is a robust model for understanding the relationship between personality and behaviors (Poropat, 2009). This model presents five personality traits: extroversion, agreeableness, conscientiousness, emotional stability, and openness to experience.

#### 2.1.1 Extroversion and burnout

Extroversion describes a socially outgoing individual, who enjoys engaging in conversation and is active. This description corresponds with lively sociability, as defined by Eysenck (Mccrae and Costa, 1989). Extroverted individuals can adjust to different situations and rapid changes (Rao and Pradhan, 2007). Jung (1936) described extroversion as the inclination to find enjoyment in noise and activity, demonstrating an externally focused individual with a wide social circle. These individuals demonstrate focused problem-solving strategies, engage in rational activities, and thrive on positive feedback. These qualities are related to burnout (Bakker et al., 2006). Additionally, De la Fuente-Solana et al. (2021) reported a negative relationship between extroversion and burnout. Similarly, Narang et al. (2022) in a study of medical practitioners reported a negative relationship between extroversion and burnout Therefore, it is assumed that:

*Hypothesis 1: There is a negative relationship between extroversion and burnout.*

#### 2.1.2 Agreeableness and burnout

Digman (1990), describes agreeableness as “seems tepid for a dimension that appears to involve the more human aspects of humanity.” This factor primarily pertains to interpersonal orientation (Pervin, 2001). On one end of the spectrum, this trait encompasses characteristics such as caring, cooperativeness, and selflessness; while on the opposite end, it may manifest as indifference, callousness, and viciousness (Mccrae and Costa, 1989; Digman, 1990; Haslam, 2007). Individuals who score high on agreeableness typically demonstrate trusting behavior, pleasure, and cooperation with others, while individuals who score low on agreeableness are quarrelsome (Digman, 1990; Zellars et al., 2000). The caring factor of this trait often results in effective customer management, handling existing frustrations, and consequently leading to a lower likelihood of depression and enhanced personal accomplishment (Digman, 1990). Additionally, a study by Dionigi (2020) reported a negative relationship between agreeableness and burnout. The study of Montag et al. (2022) also reported this negative relationship. Based on the aforementioned qualities and related studies of agreeableness, we assume that:

*Hypothesis 2: There is a negative relationship between agreeableness and burnout.*

#### 2.1.3 Conscientiousness and burnout

Individuals exhibiting high scores in this trait are described as well-organized, efficient, goal-striving, thorough, diligent, and achievement-oriented (McCrae and Costa, 1987; Haslam, 2007). Conversely, individuals with low scores on this trait tend to be impulsive, careless towards responsibilities, and disorganized (Haslam, 2007). Those high in conscientiousness tend to prioritize self-efficacy and goal-directed behaviors and are result-oriented. These factors may lead to depersonalization, however, goal-directed behavior and commitment to accomplishment often result in professional accomplishment (Zellars et al., 2000). High scorers in conscientiousness often demonstrate problem-solving strategies and display less use of avoidance and self-blaming strategies (Bouchard et al., 2004). Thus, it appears that high conscientiousness scorers experience less burnout (Zellars et al., 2000; Vukmirovic et al., 2020; Wang et al., 2022). Therefore, we assume the following:

*Hypothesis 3: There is a negative relationship between conscientiousness and burnout.*

#### 2.1.4 Emotional stability and burnout

Emotional stability is the desirable personality trait that refers to an individual’s ability to maintain emotional stability (Mccrae and Costa, 1989). Individuals with emotional stability can overcome difficult circumstances, manage challenges, and remain productive and competent. Individuals perceived as neurotic (low emotional stability) tend to fixate on the negative aspects of any transitions in their work or life environments (Swider and Zimmerman, 2010). In comparison to neurotic individuals, those with a more stable emotional personality are more likely to experience a higher degree of workplace satisfaction and may demonstrate improved progress (Jia et al., 2013). Additionally, Vigouroux and Scola (2018) reported a negative relationship between emotional stability and burnout. Given these characteristics of emotionally stable individuals, we hypothesized that:

*Hypothesis 4: There is a negative relationship between emotional stability and burnout.*

#### 2.1.5 Openness to experience and burnout

Taylor and De Bruin (2006), defined this trait as embodying curiosity regarding their words, an inclination towards experientially rich lives, and an openness to new ideas and unconventional values. Open people actively seek out new experiences, contemplate new ideas, comprehend new ideas, and appreciate diverse interests. Conversely, individuals scoring low on openness to experience tend to exhibit conventional and conservative tendencies, displaying limited interests and a reluctance to question traditional values or modes of thinking. Generally, these individuals are more creative and intelligent when compared to others. These individuals seem to have novel thinking skills, a wide range of interests and amplified imaginations. Therefore, they can identify stressors in the environment and view them as challenges because they enjoy trying new experiences. Consequently, they minimize the chances of emotional exhaustion and foster greater belief in personal accomplishment (Zellars et al., 2000). Additionally, a study by Bhowmick and Mulla (2021) reported a negative relationship between personality and burnout. Therefore, we assume the following:

*Hypothesis 5: There is a negative relationship between openness to experience and burnout.*

### 2.2 Digital competency

Digital competency is necessary in the current technological era. According to the Council of the EU, digital competency “involves the safe and critical use of the technologies of the information society for work, leisure, and communication” (Røkenes and Krumsvik, 2014). This societal presence extends to the educational sector, where teaching faculties have an unprecedented array of technological resources to execute their professional teaching plans (Falloon, 2020). Specifically, digital teaching competence involves acquiring a specific set of skills, knowledge, and attitudes essential for the technical, pedagogical, and didactic integration of information and communication technologies (ICT) within educational settings (Cabero-Almenara et al., 2021).

Studies have highlighted the specific benefits of using and integrating various technologies in medical education and incorporating digital competencies into allied health education (Moro et al., 2019; Foadi and Varghese, 2022). These technologies facilitate allied health students in staying current with the dynamic and continuously evolving nature of allied health education (Montero Delgado et al., 2020). The possession of digital competencies by faculty members in allied health plays a vital role in improving the quality of medical education. Therefore, the digital competencies of allied health teachers are considered pivotal competencies in the current technological age (Bublii et al., 2022).

#### 2.2.1 Moderating role of digital competencies

The JD-R theory divides job characteristics into two categories: job demands and resources, which are prevalent across various job types. Job resources can be defined as social, psychological, physical, and organizational aspects of work aimed at reducing the impact of job demands (Bakker et al., 2007). Job demands refer to the social, psychological, physical, and organizational aspects of a job that utilize job resources to complete work-related tasks for achieving organizational objectives (Bakker et al., 2014). JD-R theory states that job demands and resources are the initiators of employees’ health impairment and motivation. JD-R theory further states that personal resources may buffer the impact of job demands and act as moderators by minimizing their negative impact on job demands. In this study, burnout was a negative psychological state experienced by teachers and its relationship with their personalities. According to the JD-R theory, personal resources can moderate the negative psychological state of employees. Therefore, this study evaluated teachers’ digital competence as a personal job resource and examined the moderating role of the relationship between teachers’ personalities and burnout. Existing studies have examined the moderating role of digital competence in the relationship between employee behaviors and job outcomes and reported it as a potential moderator (Li et al., 2021; Aldhaen, 2023). Based on this theoretical foundation, we hypothesize the following:

*Hypothesis 6: The negative relationship between extroversion and burnout will be moderated by teachers’ digital competence.*

*Hypothesis 7: The negative relationship between agreeableness and burnout will be moderated by teachers’ digital competence.*

*Hypothesis 8: The negative relationship between conscientiousness and burnout will be moderated by teachers’ digital competence.*

*Hypothesis 9: The negative relationship between emotional stability and burnout will be moderated by teachers’ digital competence.*

*Hypothesis 10: The negative relationship between openness to experience and burnout will be moderated by teachers’ digital competence.*

## 3 Methods

### 3.1 Sample size and data collection procedures

Allied health institution educators from Pakistan were asked to provide information through self-reported questionnaires. The questionnaires were distributed in English because the participants were fluent in English. Therefore, the questionnaire was not translated into the local language. A total of 400 questionnaires were distributed for data collection. In total, 369 questionnaires were returned in a single wave, of which 35 were incomplete. Consequently, 334 questionnaires were included in the data analysis, yielding a response rate of 83%. The participants were asked to provide information on the big five personality traits, teachers’ digital competence, and burnout. A single-factor Harman’s test was executed to check for biases in the data and found a value of 21%, below the threshold of 50%. There was no risk of a common method bias. In addition, demographic data about the participants’ age, sex, and qualifications were recorded. Table 1 shows the demographic characteristics of the participants.

**Table 1:** Demographic characteristics of participants.

	**Frequency**	**Percentage**
**Sex**		
M	173	51.8
F	161	48.2
**Age**		
25 or below	38	11.4
26–30	81	24.3
31–35	77	23.1
36–40	65	19.5
41–45	55	16.5
46 or above	18	5.4
**Education**		
Bachelors	37	11.1
Masters	222	66.5
M. Phil	59	17.7
Postgraduates.	16	4.8

### 3.2 Measures

#### 3.2.1 Big-five personality traits

Responses to the big-five personality traits were rated on a 5-point Likert scale as follows: 1 = very inaccurate, 2 = inaccurate, 3 = neither accurate nor inaccurate, 4 = accurate, and 5 = very accurate.

We measured the personality traits using 50 items of IPIP developed by Goldberg (1992). Every personality trait is measured by a sub-scale of 10 items. The coefficient alpha was 0.84 for the source scale. These items are available at https://ipip.ori.org/.

#### 3.2.2 Digital competence

Digital competence was measured using the DigCompEdu 22-item instrument. This instrument measures six competence dimensions: professional engagement, digital resources, teaching and learning, assessment, empowering learners, and facilitating learner’s digital competence. Each item is measured on a 5-point Likert scale. The participants rated their teaching practices by choosing one of the five options. These options are progressively organized, indicating the overall progression logic of digital competence using an internal scoring system. This system included no commitment (0-point), partial knowledge (1-point), occasional use (2-point), increased use (3-point), and comprehensive use (4-point). This instrument was deemed fit for all teachers, with an excellent internal consistency of 0.91 (Ghomi and Redecker, 2019). The items in this instrument are available at https://bit.ly/2ZyfyQR.

#### 3.2.3 Burnout

The teachers’ burnout scale comprised 22 items from an educator burnout inventory developed by Maslach (1996), using 9 items for emotional exhaustion, 5 items for depersonalization, and 8 items for self-accomplishment. Responses were rated on a 5-point Likert scale ranging from 1 = strongly disagree to 5 = strongly agree. An example item for burnout was “I feel emotionally drained from my work.” The reliability of burnout on Cronbach’s alpha was 0.82 in a previous study (Christina Maslach, 1981), which is above the threshold of 0.70. Therefore, the scale related to burnout was reliable for data collection.

### 3.3 Control variables

Previous studies reported that age, sex, and education have significant effects on burnout (Schaufeli and Enzmann, 1998; Ahola and Honkonen, 2006). Therefore, in this study, we opted to test the demographic variable as the independent variable by performing a one-way analysis of variance. The results revealed that the three demographic variables were not significant.

## 4 Results and analysis

Data were analyzed using IBM SPSS Statistics for Windows, Version 20.0 (Armonk, NY: IBM Corp). Table 2 shows the descriptive, bivariate, and reliability (coefficient) estimates for all measures. All zero-order bivariate correlations were based on expected directions.

**Table 2:** Descriptive and correlation.

**Variables**	**Mean**	**S.D.**	**1**	**2**	**3**	**4**	**5**	**6**	**7**
1. Extroversion	3.35	0.73	1						
2. Agreeableness	3.37	0.70	0.428**	1					
3. Conscientiousness	3.32	0.94	0.266**	0.424**	1				
4. Emotional stability	3.40	0.99	−0.057	−0.053	0.125*	1			
5. Openness to experience	3.32	0.84	0.284**	0.388**	0.353**	0.087	1		
6. Digital Competencies	2.27	0.83	0.182**	0.196**	0.194**	0.082	0.290**	1	
7. Teachers’ burnout	3.41	0.76	−0.221**	−0.243**	−0.119*	−0.128*	−0.207**	−0.421**	1

***Correlation is significant at the 0.001 level (two-tailed). **Correlation is significant at the 0.01 level (two-tailed). *Correlation is significant at the 0.05 level (two-tailed).

### 4.1 Reliability and validity analysis

To validate the analysis, we performed a confirmatory factor analysis on the dataset. For this purpose, AMOS v23 was used. Table 3 summarizes the reliability and validity of the analyses.

**Table 3:** Reliability and validity analysis.

**Variable**	**α**	**CR**	**AVE**	**MSV**	**1**	**2**	**3**	**4**	**5**	**6**	**7**
DTC	0.94	0.936	0.512	0.156	**(0.716)**						
BO	0.92	0.935	0.506	0.156	−0.395***	**(0.712)**					
OP	0.90	0.834	0.503	0.147	0.309***	−0.163*	**(0.709)**				
EX	0.83	0.780	0.544	0.083	0.170*	−0.255***	0.257***	**(0.737)**			
AG	0.80	0.809	0.519	0.135	0.109†	−0.172**	0.270***	0.284***	**(0.720)**		
CON	0.94	0.864	0.615	0.147	0.163**	−0.099	0.383***	0.288***	0.368***	**(0.784)**	
ES	0.93	0.841	0.570	0.020	0.093	−0.141*	0.038	−0.109	0.040	0.123†	**(0.755)**

DTC, digital competence; BO, burnout; OP, openness to experience; EX, extroversion; AG, agreeableness; CON, conscientiousness; ES, emotional stability, Reliability estimates in parentheses; *p < 0.05, **p < 0.01, ***p < 0.001. The square root of AVE is given in parentheses in bold numbers.†α = Cronbach alfa.

The α value for all variables in Table 3 is between the minimum and maximum threshold values of 0.70 and 0.95, respectively, revealing reliable internal consistency of the scale. The average variance extracted (AVE) is an appropriate measure for testing the convergent validity of a scale. For this purpose, the value of AVE should be greater than 0.50, leaving no doubt of convergent validity (Fornell and Larcker, 1981). In the current study, the AVE values of all constructs were greater than 0.50. The mean shared variance (MSV) is a good representative of the discriminant validity. If the MSV was less than half of the AVE, the discriminant validity of the scale items was considered valid (Carver and Glass, 1976). In the current study, the MSV of all constructs was less than half the AVE, confirming the validity of the scale. The second criterion of discriminant validity according to Fornell and Larcker (1981) is that if the square root of the AVE is greater than the correlation of the latent variable, it confirms the absence of discriminant validity issues in the scale. In this study, the square root of the AVE of all the constructs was greater than the correlation of the latent variable. The values presented in Table 3 reveal that the scales present no concerns regarding reliability and validity (Gaskin and Lim, 2016).

### 4.2 Model fit analysis

**Figure 1:**
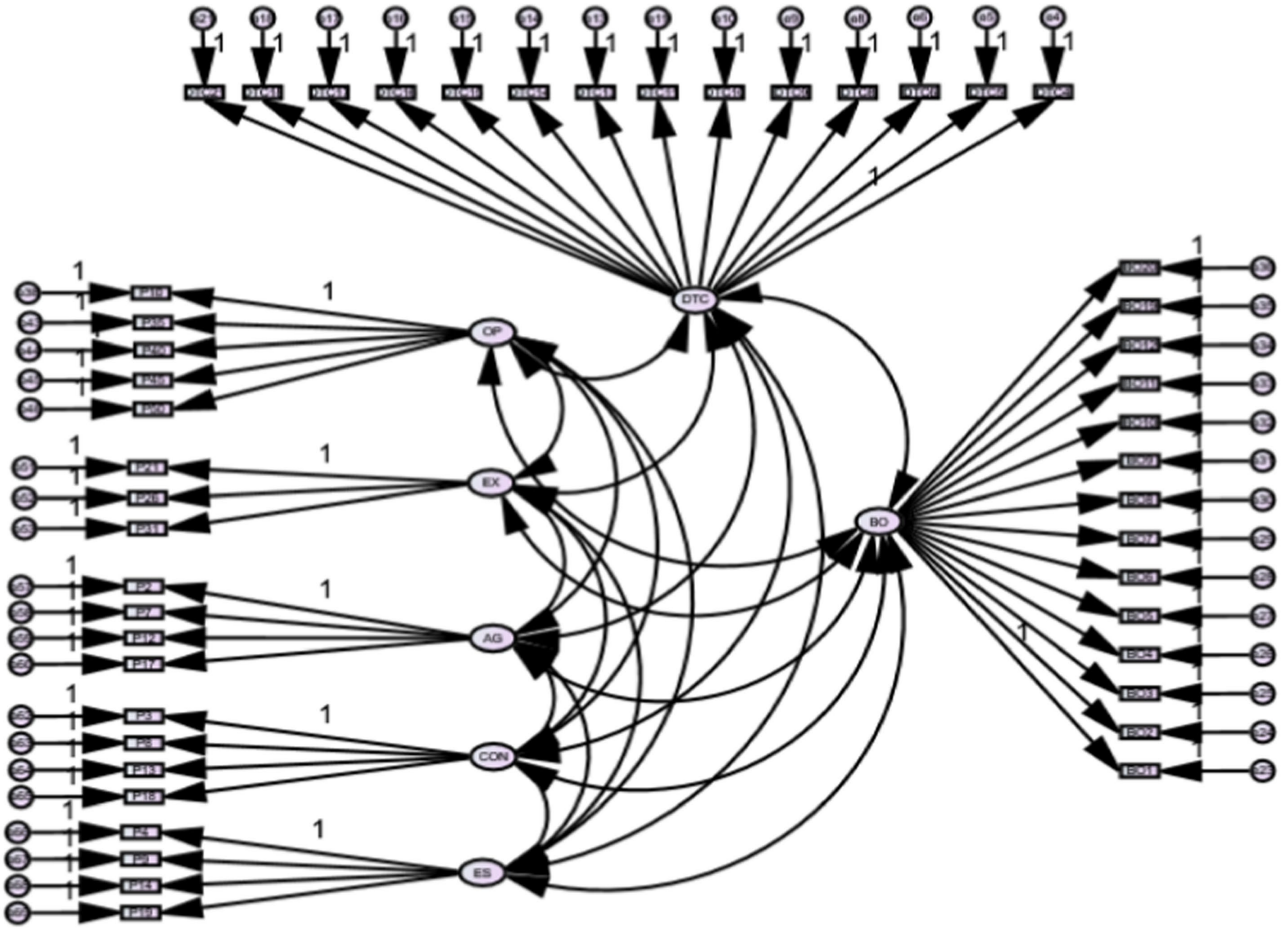
Measurement model fit analysis. DTC, digital competence; BO, burnout; OP, openness to experience; EX, extroversion; AG, agreeableness; CON, conscientiousness; ES, emotional stability.

Table 4 summarizes the goodness-of-fit statistics of the measurement model. The value of the relative chi-square was 1.510, between the threshold of 1–3. The comparative fit index value was 0.932, below the threshold of 0.95. The Root Mean Square Error of Approximation (RMSEA) achieved a value of 0.046 below the threshold value of 0.06. The standard Root Mean Square Residual (SRMR) achieved a value of 0.039, and the value of PClose was >0.05, achieving a value of 1.00. All values listed in Table 4 indicate that the model fit is acceptable (Gaskin and Lim, 2016). See Figure 1.

**Table 4:** Model fit analysis.

Goodness-of-fit statistics for measurement model		
	Guidelines	Achieved
Chi-Square (CMIN)	Significant	1598.635
DF	NA	1,059
CMIN/DF	<3	1.510
CFI	<0.95	0.932
RMSEA	<0.06	0.046
SRMR	<0.05	0.039
PClose	>0.05	1.00

### 4.3 Direct effects

To test the direct effect of personality traits on teachers’ burnout, we conducted a simple linear regression analysis for Hypotheses 1 to 5. Extroversion was used as the independent variable and burnout as the dependent variable. The results show that extroversion is negatively related to burnout (β = −0.230, *p* < 0.001). Our findings suggest that teachers scoring high in extroversion tend to demonstrate a lower likelihood of experiencing burnout; thus, Hypothesis 1 is supported. To test Hypothesis 2, we used agreeableness as the independent variable and burnout as the dependent variable. The results showed a negative relationship between agreeableness and burnout (*β* = −0.262, *p* < 0.001). Our findings suggest that teachers with high agreeableness scores have a lower likelihood of experiencing burnout; thus, Hypothesis 2 is supported.

To test Hypothesis 3, we used conscientiousness as the independent variable and burnout as the dependent variable. The results show that conscientiousness is negatively related to burnout (*β* = −0.096, *p* < 0.01). Our findings suggest that teachers with high levels of conscientiousness tend to demonstrate fewer burnout symptoms. Therefore, Hypothesis 3 is supported by our results. To test Hypothesis 4, we used emotional stability as an independent variable and burnout as a dependent variable. The results showed a negative relationship between emotional stability and burnout (*β* = −0.081, *p* < 0.01). These findings suggest that teachers who scored high on emotional stability tended to show fewer symptoms of burnout. Therefore, Hypothesis 4 was supported. Openness to experience was used as the independent variable and burnout as the dependent variable to test Hypothesis 5. The results show a negative relationship between openness to experience and burnout (*β* = −0.188, *p* < 0.01). Our findings reveal that teachers who score high on openness to experience tend to show fewer symptoms of burnout, supporting Hypothesis 5.

### 4.4 Moderating effects

**Figure 2:**
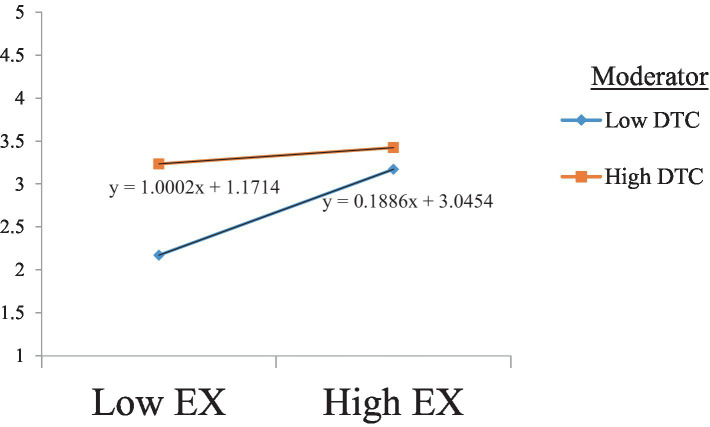
The moderating effect of DTC on the relationship between Ex and Bo. DTC, digital competence; EX, extroversion.

In this study, Hayes’ PROCESS macro v2.16.3 was used to test the moderating effect of teachers’ digital competence on the relationship between personality traits and burnout. Model 1 tested the moderating effect of teachers’ digital competence on the negative relationship between extroversion and burnout. The results presented in Table 5 show a significant moderating effect, with no zero between the LLCI and ULCI. This finding is consistent with Hypothesis 6. Our findings suggest that teachers scoring high in digital competence tend to demonstrate fewer burnout symptoms, even with a low level of extroversion. The slope analysis further confirmed these results, as shown in Figure 2.

**Table 5:** Moderation effect of DTC on the relationship between Ex and Bo.

	**Coeff**	**se**	***t***	***p***	**LLCI**	**ULCI**
**Constant**	3.2355	0.4971	6.5082	0.0000	2.2575	4.2134
**DTC**	0.3293	0.2142	1.5375	0.1251	−0.0920	0.7506
**EX**	0.2973	0.1466	2.0284	0.0433	0.0090	0.5857
**int_1**	−0.2030	0.0615	−3.3000	0.0011	−0.3241	−0.0820

Outcome variablem Bo; DTC, Digital competence, EX, extroversion.

**Figure 3:**
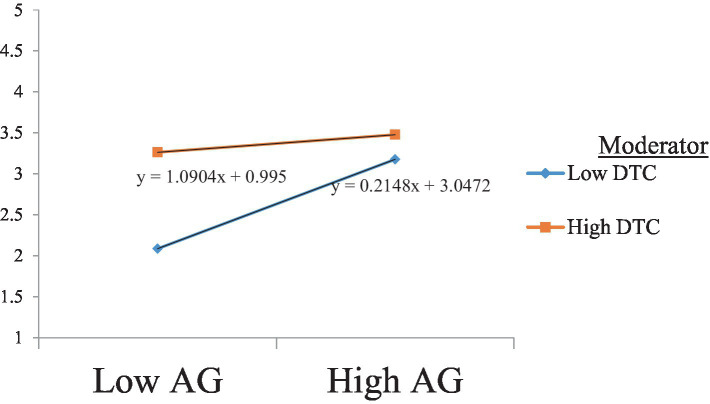
The moderating effect of DTC on the relationship between Ag and Bo. DTC, digital competence; AG, agreeableness.

Our Hypothesis 7 suggests that the negative relationship of agreeableness to burnout is moderated by teachers’ digital competence such that this negative relationship is strengthened at a high level of teachers’ digital competence. The results presented in Table 6 show the significant moderating effect of teachers’ digital competence, with no zero between the LLCI and ULCI. These results are consistent with Hypothesis 7. The slope analysis further confirms these results, as shown in Figure 3.

**Table 6:** Moderation effect of DTC on the relationship between Ag and Bo.

	**Coeff**	**se**	***t***	***p***	**LLCI**	**ULCI**
**Constant**	3.1749	0.5033	6.3087	0.0000	2.1849	4.1649
**DTC**	0.3692	0.2092	1.7650	0.0785	−0.0423	0.7807
**AG**	0.3255	0.1519	2.1425	0.0329	0.0266	0.6243
**int_1**	−0.2187	0.0615	−3.5529	0.0004	−0.3398	−0.0976

Outcome variable, Bo; DTC, digital competence; AG, agreeableness.

**Figure 4:**
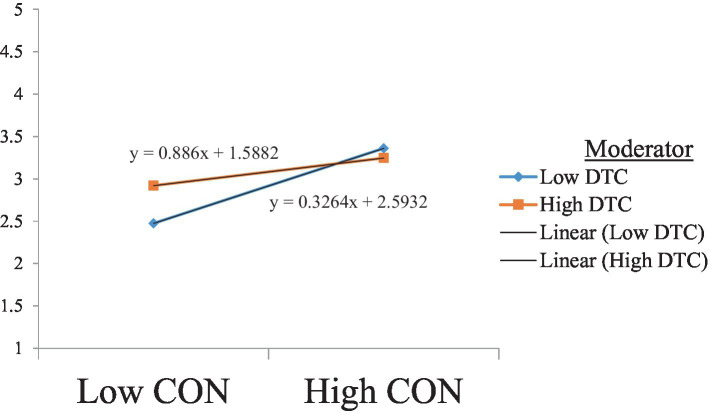
The moderating effect of DTC on the relationship between Con and Bo. DTC, digital competence; CON, conscientiousness.

The negative relationship between conscientiousness and burnout is moderated by teachers’ digital competence, such that teachers who score high in digital competence tend to present low burnout symptoms even with low conscientiousness. The results presented in Table 7 show a significant moderating effect of teachers’ digital competence, with no zero between the LLCI and ULCI. These results supported Hypothesis 8. Figure 4 shows the slope analysis confirming these findings.

**Table 7:** Moderation effect of DTC on the relationship between Con and Bo.

	**Coeff**	**se**	***t***	***p***	**LLCI**	**ULCI**
**Constant**	3.2949	0.3757	8.7711	0.0000	2.5560	4.0339
**DTC**	0.0828	0.1527	0.5420	0.5882	−0.2176	0.3831
**CON**	0.3023	0.1125	2.6866	0.0076	0.0810	0.5237
**int_1**	−0.1397	0.0440	−3.1760	0.0016	−0.2262	−0.0532

Outcome variable, Bo; DTC, digital competence; CON, conscientiousness.

**Figure 5:**
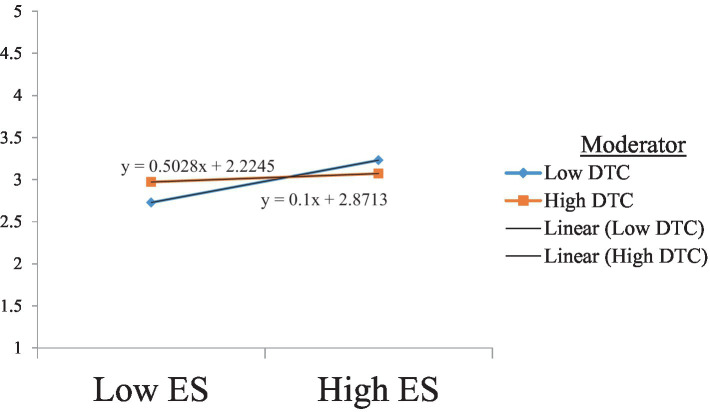
The moderating effect of DTC on the relationship between Es and Bo. DTC, digital competence; ES, emotional stability.

Hypothesis 9 suggests that the negative relationship between emotional stability and burnout is moderated by teachers’ digital competence, such that teachers who score high in digital competence experience less burnout. The results presented in Table 8 show a significant moderating effect of teachers’ digital competence, with no zero between LLCI and ULCI; therefore, our findings support Hypothesis 9. The slope analysis in Figure 5 confirms our findings.

**Table 8:** Moderation effect of DTC on the relationship between Es and Bo.

	**Coeff**	**se**	***t***	***p***	**LLCI**	**ULCI**
**Constant**	3.5443	0.3784	9.3667	0.0000	2.7999	4.2886
**DTC**	0.0428	0.1630	0.2627	0.7930	−0.2778	0.3635
**ES**	0.2041	0.1032	1.9768	0.0489	0.0010	0.4072
**int_1**	−0.1185	0.0438	−2.7077	0.0071	−0.2046	−0.0324

Outcome variable, Bo; DTC, digital competence; ES, emotional stability.

**Figure 6:**
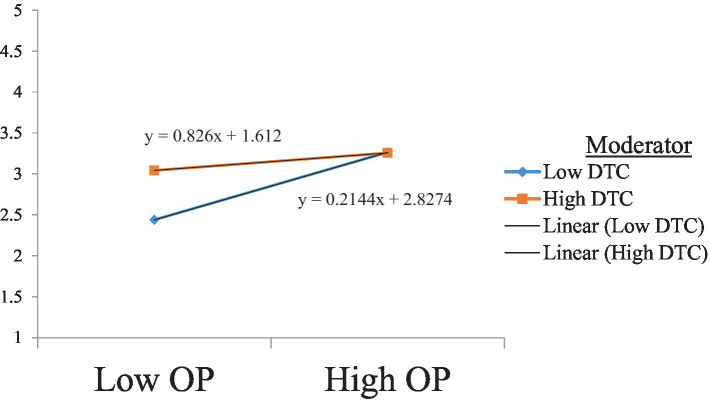
The moderating effect of DTC on the relationship between Op and Bo. DTC, digital competence; OP, openness to experience.

Hypothesis 10 suggests that the negative relationship between openness to experience and burnout is moderated by teachers’ digital competence such that the relationship is strengthened with a higher level of teachers’ digital competence. The results presented in Table 9 show a significant moderating effect of teachers’ digital competence on the negative relationship between openness to experience and teachers’ burnout, with no zero between LLCI and ULCI. This finding is consistent with Hypothesis 10. Furthermore, the slope analysis confirmed these findings (Figure 6).

**Table 9:** Moderation effect of DTC on the relationship between OP and BO.

	**Coeff**	**se**	***t***	***p***	**LLCI**	**ULCI**
**Constant**	3.3943	0.3883	8.7410	0.0000	2.6304	4.1582
**DTC**	0.1494	0.1673	0.8926	0.3727	−0.1798	0.4786
**OP**	0.2596	0.1176	2.2066	0.0280	0.0282	0.4910
**int_1**	−0.1528	0.0480	−3.1824	0.0016	−0.2473	−0.0584

Outcome variable, Bo; DTC, digital competence; OP, openness to experience.

## 5 Discussion

This study was conducted to examine the relationship between personality and teachers’ burnout stress in Pakistan to deepen our understanding of this relationship in a non-Western cultural context. This study also examined whether teachers’ digital competence could be used to counteract burnout effects.

Data collected from 334 teachers from allied health institutions revealed that extroversion negatively affects burnout. In other words, teachers with highly extroverted personality traits experience fewer burnout symptoms. Our findings are consistent with those of De la Fuente-Solana et al. (2021) reported a negative relationship between extroversion and burnout, specifying the protecting capacity of extroversion against burnout and these individuals experienced fewer stress symptoms. The findings of current research are also consistent with those of Prins et al. (2019) who reported a negative relationship between extroversion and burnout among medical specialists. This study’s findings reported that the personality trait of agreeableness negatively influenced burnout. These findings are supported by Swider and Zimmerman (2010), who reported that agreeable individuals receive positive feedback, find positivity in their workplace activities, and are less exposed to burnout stress syndrome (John and Srivastava, 1999; Graziano et al., 2007). Additionally, Dionigi (2020) in a study of healthcare workers, reported a negative relationship between agreeableness and burnout. According to our findings, teachers who scored high on conscientiousness were less likely to experience burnout. These findings aligned with our hypotheses. Individuals who score high on conscientiousness focus on achieving job-related goals and remain positive in the workplace (Zellars et al., 2000). These findings are also consistent with the traditional view that conscientious individuals are high performers in various job contexts (Vukmirovic et al., 2020). The personality trait of emotional stability is negatively related to teacher burnout, and teachers with high emotional stability experienced less burnout at work. These findings were consistent with those of previous studies. For example, Swider and Zimmerman (2010) reported in their meta-analysis that individuals with low emotional stability (neurotic) are more likely to experience burnout. Jia et al. (2013) reported that people with high emotional stability can deal with work-related stress and remain positive, productive, and happy at work. Similarly, Vigouroux and Scola (2018) reported that emotional stability prevents individuals from experiencing burnout stress. In the current study, openness to experience negatively related to burnout, where teachers with high scores in this domain were less vulnerable to burnout. These findings were consistent with those of previous studies. These individuals perceive work difficulties as opportunities for personal growth because of their open-mindedness and intellectual curiosity (Kim et al., 2019). Therefore, they have fewer chances of experiencing burnout and are significantly associated with problem-focused strategies (Strutton et al., 1995). Additionally, Bhowmick and Mulla (2021) reported a negative relationship between openness to experience and burnout. The above-mentioned findings of the relationships between all five personality traits and burnout stress syndrome are consistent with those of Vukmirovic et al. (2020), who conducted their study on medical faculty staff. Furthermore, the findings of the current study about personality traits and burnout are in line with a recent systematic literature review of Angelini (2023b). therefore, among many other professional groups, teachers’ personalities are related to burnout syndrome, and medical teaching staff in allied health institutions are no exception.

Furthermore, we explored the moderating role of teachers’ digital competence on the relationship between personality traits and burnout. Teachers’ digital competence moderated the negative relationship between burnout and extroversion, agreeableness, conscientiousness, emotional stability, and openness to experience. Findings supporting the moderating role of digital competencies as a job resource and potential moderator align with existing studies (Jia et al., 2013; Triyono et al., 2023).

## 6 Conclusion

This study examined the direct effect of personality traits on teacher burnout in the non-Western culture of Pakistan. Furthermore, it evaluated the buffering role of digital competence as a personal job resource. First, the current study concludes that personality is an intra-individual antecedent related to teachers’ job burnout in allied health institutions. Second, the direct relationship between personality traits and teacher burnout is evident in non-Western cultures, such as Pakistan. Therefore, the generalizability of this direct relationship can be extended to South Asian cultural contexts. Third, this study concludes that digital competence may be recognized as a personal job resource and potential moderator of burnout. This capacity for digital competence can assist teachers in managing work-related stress in digital working environments. Theoretically, this study has forged a new path for digital competence researchers to explore, evaluate, and enhance complex models. Digital competence has merged as a new job resource in this digital era, which not only possesses the capacity to mitigate organizational job demands but also acts as a buffer among individual differences and burnout stress. The current study concludes that digital competence as a job resource in the digital work environment may enhance the JD-R resource theory in a modern working digital environment. However, further research is required in a variety of cultural settings.

### 6.1 Practical and theoretical implication

This study had several theoretical and practical implications. Managers and policymakers should be aware of the relationship between personality traits and teacher burnout. Certain personality traits can either cause or prevent stress for teachers. Therefore, training and curricula can identify and promote favorable personality traits for the teaching profession. Longitudinal studies have recommended the identification of favorable personality traits and suggested that personality-related changes occur during young adulthood (Roberts and Jackson, 2008). Furthermore, this study established an empirical basis for digital competence as a moderator. Teachers’ digital competence was established as a resource for coping with job burnout among allied health institution educators. Therefore, future teachers should acquire digital competencies for improved performance, and advanced digital competency courses should be included in their curriculum. According to a literature review by Zhao et al. (2021), future teachers should acquire basic digital competencies in their teaching training programs. For current teachers, comprehensive training should be provided to integrate the use of digital tools and create more effective teaching methods.

### 6.2 Strengths, limitations, and future research directions

This study has several strengths. First, it responds to the need for studies in non-Western cultures to test theories in different cultural settings (Henrich et al., 2010; Klassen et al., 2018). Specifically, personality and burnout research has been recommended to be conducted in different countries (Kim et al., 2019). The current study was conducted in Pakistan, a different culture from North America and Europe. Exploring the predominant and moderating effects in this setting elevates our understanding and confirms the external validity of research that has primarily used samples from Western countries. Second, teachers’ digital competencies have not been studied as moderators in the relationship between individual factors and burnout stress syndrome. This tool proved to be an excellent resource for mitigating burnout effects. This expands our understanding of the JD-R Model and provides digital competence as a tool for intervention in burnout stress, adding a valuable contribution to the existing literature on JD-R theory.

This study has some limitations. First, the data were collected from allied health institution educators. Therefore, this limitation should be considered before generalizing the results to the entire teacher community and allied health professionals. Second, we used a cross-sectional design for the data collection. A longitudinal design can comprehensively examine the relationship between personality traits. Future studies should use a longitudinal design with diverse sample sizes to overcome these limitations. The relationship between job demands and job outcomes should be examined to evaluate the moderating role of teachers’ digital competence. Future research can examine and replicate this research model in different cultures and other populations. The potential role of digital competence as a personal job resource can be examined in future research on the relationship between work environment stress factors and employees’ work engagement or burnout.

## Data availability statement

The raw data supporting the conclusions of this article will be made available by the authors, without undue reservation.

## Ethics statement

Ethical review and approval was not required for the study on human participants in accordance with the local legislation and institutional requirements. Written informed consent from the participants was not required to participate in this study in accordance with the national legislation and the institutional requirements.

## Author contributions

ZH: Writing – original draft, Conceptualization, Data curation, Investigation, Methodology, Software, Validation, Writing – review & editing. CC: Conceptualization, Funding acquisition, Project administration, Supervision, Visualization, Writing – review & editing. MS: Data curation, Formal analysis, Visualization, Writing – review & editing. NH: Data curation, Visualization, Writing – review & editing. FC: Data curation, Visualization, Writing – review & editing.
