# Supplementary material for: Personality and teachers’ burnout stress: exploring the digital competence as personal job resource in allied health institutions
Source: Front Psychol. 2024 May 9;15:1334371. doi: 10.3389/fpsyg.2024.1334371 (PMC11112098; doi:10.3389/fpsyg.2024.1334371)
Supplement: Supplementary file 1 [file Data_Sheet_1.pdf]

Work Opinion Survey

Dear Respondent! I am a PhD Scholar in Guangzhou University, China. My research is about “Personality and teachers’ burnout stress”. This study is a purely academic-base activity and your responses will be interpreted on generalized basis. All the responses will be kept in confidence. Your participation is highly appreciable, if you find any conflict of interests, you are free to withdraw your participation from this study. I am really thankful for your cooperation.

Section: A

1. Gender

1.Male

2. Female

2. Age (in years).

1. 25 or below	
2. 26 -- 30	
1. 31 – 35	
2. 36 -- 40	
3. 41 – 45	
4. 46 or above	

Qualification (last degree).

1.Bachelor	
2.M. Phil	
3. Masters	
4.Postgraduate.	

Section: B

Describe yourself as you generally are now, not as you wish to be in the future. Describe yourself as you honestly see yourself, in relation to other people you know of the same sex as you are, and roughly your same age. So that you can describe yourself in an honest manner, your responses will be kept in absolute confidence. Indicate for each statement whether it is 1. Very Inaccurate, 2. Moderately Inaccurate, 3. Neither Accurate nor Inaccurate, 4. Moderately Accurate, or 5. Very Accurate as a description of you.

**Statements**	Very Inaccurate	Moderately Inaccurate	Neither Accurate Nor Inaccurate	Moderately Accurate	Very Accurate
1. I am the life of the party.					
2. I feel little concern for others.					
3. I am always prepared.					
4. I get stressed out easily.					
5. I have a rich vocabulary.					
6. I don't talk a lot.					
7. I am interested in people.					
8. I leave my belongings around.					
9. I am relaxed most of the time.					
10. I have difficulty understanding abstract ideas.					
11. I feel comfortable around people.					
12. I insult people.					
13. I pay attention to details.					

14. I worry about things.					
15. I have a vivid imagination.					
16. I keep in the background.					
17. I sympathize with others' feelings.					
18. I make a mess of things.					
19. I seldom feel blue.					
20. I am not interested in abstract ideas.					
21. I start conversations.					
22. I am not interested in other people's problems.					
23. I get chores done right away.					
24. I am easily disturbed.					
25. I have excellent ideas.					
26. I have little to say.					
27. I have a soft heart.					
28. I often forget to put things back in their proper place.					
29. I get upset easily.					
30. I do not have a good imagination.					
31. I talk to a lot of different people at parties.					
32. I am not really interested in others.					
33. I like order.					
34. I change my mood a lot.					
35. I am quick to understand things.					
36. I don't like to draw attention to myself.					
37. I take time out for others.					
38. I shirk my duties.					
39. I have frequent mood swings.					
40. I use difficult words.					
41. I don't mind being the center of attention.					
42. I feel others' emotions.					
43. I follow a schedule.					
44. I get irritated easily.					
45. I spend time reflecting on things.					
46. I am quiet around strangers.					

47. I make people feel at ease.					
48. I am exacting in my work.					
49. I often feel blue.					
50. I am full of ideas.					

Section: C

This section is about your use of digital technology and digital tool in your daily teaching activities. Please read the statement given and choose the one option among (A, B, C, D, E) that best reflects your current practice.

1. I systematically use different digital channels to improve communication with students, families and my colleagues. (emails, WhatsApp etc.)

A	I rarely use digital communication channels
B	I use basic digital communication channels. (example, email)
C	I combine different communication channels. (class blog, center website).
D	I systematically select, adjust and combine different digital communicate channels
E	I reflect, discuss and proactively develop my communication strategies

2. I use digital technologies to work with my colleagues inside and outside my educational organization

A	I rarely have the opportunity to collaborate with other colleagues
B	Sometimes I exchange materials with colleagues. (via pen drive, email)
C	Among colleagues, we work together in collaborative environments or use shared units.
D	I exchange ideas and materials with other teachers outside my organization.
E	I create materials collaboratively with other teachers in an online network

3. I actively develop my digital teaching competence.

A	I rarely have time to work on my teaching digital competence.
B	I improve my competence through reflection and experimentation
C	I use different resources to develop my digital teaching competence
D	I discuss with my colleagues for using digital technologies to innovate and improve educational practice.
E	I create materials collaboratively with other teachers in an online network.

4. I participate in online training courses. For example: online administration courses, MOOCs, webinars.

A	It's something I haven't considered yet.
B	Not yet, but I'm interested in it.
C	He participated in 1 or 2 online teacher training courses
D	I have participated in more than 2 online teacher training courses.
E	I frequently participate in all types of online courses that improve my training as a teacher

5. I use different internet sites (web pages) and search strategies to find and select a wide range of digital resources.

A	I rarely use the internet to find resources.
B	I use search engines (e.g.Google) and/or educational platforms to find educational resources.
C	I evaluate and select the digital resources I find based on their suitability for my group of students.
D	I compare resources using a series of criteria relevant to my educational practice
E	I advise colleagues on appropriate digital resources and search strategies for them.

6. I create my own digital resources and modify existing ones to adapt them to my needs as a teacher.

A	I don't create my own digital resources.
B	I create activity cards with the computer and then print them.
C	I create digital slideshows. For example: Power Point, Prezi
D	I create and modify different types of digital resources.
E	I configure and adapt complex and interactive resources.

7. I securely protect sensitive content. For example: exams, grades, personal data.

A	I don't need to do that, because the educational center takes care of this.
B	I avoid storing personal data electronically
C	I protect some personal data
D	I password protect files with personal data.
E	I exhaustively protect personal data. For example: combining difficult-to-guess passwords

8. I carefully consider how, when and why to use digital technologies in class, to ensure their added value is exploited

A	I do not use or rarely use technology in class.
B	I make basic use of the available equipment. For example: audio equipment, projector,
C	I use a wide variety of digital strategies in my teaching.
D	I use digital tools to systematically improve teaching.
E	I use digital tools to implement innovative pedagogical strategies

9. I supervise my students' activities and interactions in the online collaboration environments we use

A	I do not use digital environments with my students
B	I do not monitor student activity in the online environments we use.
C	From time to time, I review them and take them into account.
D	I regularly monitor and analyze my students' online activity
E	I regularly intervene with comments to motivate or correct my students' online activity

10. When my students work in groups or teams, they use digital technologies to acquire and document knowledge

A	My students do not work in groups.
B	It is not possible for me to integrate digital technologies in group work.
C	I encourage students working in groups to search for information online or present their results in digital format.
D	When working in groups, I always ask them to use the Internet to find information and present their results in digital format.
E	My students exchange and create knowledge together in an online collaboration space. (e.g. virtual platform)

11. I use digital technologies to allow students to plan, document and evaluate their learning for themselves. For example: self-assessment tests, digital portfolio, blogs, forums.

A	It is not possible in my work environment.
B	My students reflect on their learning, but not with digital technologies
C	Sometimes I use, for example, tests for self-assessment.
D	I use a wide variety of digital tools to allow students to plan, or reflect on their learning
E	I systematically integrate different digital tools to allow students to plan, monitor and reflect on their progress

12. I use digital assessment strategies to monitor student progress.

A	I do not monitor student progress
B	I monitor student progress regularly, but not with digital means.
C	Sometimes I use digital assessment tools. For example: a questionnaire, online multiple-choice tests
D	I use a wide variety of digital tools to evaluate and monitor student progress
E	I systematically use a wide variety of digital tools to assess and monitor student progress

13. I analyze all available data to identify students who need additional support.

A	These data are not available and/or it is not my responsibility to analyze them
B	I only analyze academically relevant data. For example: performance, grades
C	I consider data about student activity and behavior to identify students who need additional support.
D	I regularly examine all available evidence to identify students who need additional support.
E	I systematically analyze the data, identify students in need of additional support, and intervene in a timely manner

14. I use digital technologies to provide effective feedback

A	Feedback is not necessary in my work environment
B	I provide feedback to students, but not in digital format
C	I sometimes use digital ways to provide feedback. For example: automatic scoring in online questionnaires,
D	I use a wide variety of digital forms of feedback
E	I systematically use digital media to provide feedback

15. When I propose digital tasks, I consider and address potential issues such as equal access to digital devices and resources; Compatibility problems or low level of digital competence of the students.

A	I don't usually propose digital tasks
B	My students have no problems with access and use of digital technology
C	I adapt the task to minimize difficulties
D	I discuss possible obstacles with students and propose solutions
E	I am flexible with digital tasks, I allow variety. For example: I adapt the task and discuss solutions,

16. I use digital technologies to offer students personalized learning opportunities. For example: assigning different digital tasks to address individual learning needs, taking into account preferences and interests

A	In my classes, all students must do the same activities
B	I provide students with additional digital resources
C	I provide optional digital activities for students who have a more advanced level or who need reinforcement.
D	Whenever possible, I use digital technologies to offer differentiated learning opportunities.
E	I systematically adapt my teaching to link to students' individual learning needs, preferences and interests

17. I use digital technologies so that students actively participate in class

A	In my classes it is not possible to actively involve the students
B	I involve students actively, but not with digital technologies
C	In my classes, I use motivating digital stimuli. For example: videos, animations, cartoons
D	My students engage with digital media in my classes. For example: online activities, games, contests, applications
E	My students systematically use digital technologies to investigate, discuss and create knowledge

18. I teach students how to evaluate the reliability of information searched online and to identify erroneous and/or biased information.

A	This is not possible in my subject or work environment.
B	From time to time, I remind them that not all information online is reliable
C	I teach them to discern between reliable and unreliable sources
D	I discuss with students how to verify the accuracy of the information
E	We thoroughly discuss how information is generated and can be distorted

19. I propose assignments that require students to use digital media to communicate and collaborate with each other or with an external audience

A	This is not possible in my subject or work environment.
B	Only rarely are my students required to communicate or collaborate online
C	My students use digital communication and cooperation mainly among themselves
D	My students use digital ways to communicate and cooperate with each other and with an external audience
E	I schedule systematic tasks that allow students to expand their communication skills by communicating with each other and with external audiences

20. I propose assignments that require students to create digital content. For example: videos, audios, photos, presentations, blogs, wikis

A	This is not possible in my subject or work environment
B	This is difficult to implement with my students.
C	Sometimes as a fun activity.
D	My students create digital content as an integral part of their learning
E	It is an integral part of their learning and I systematically increase the level of difficulty to further develop their skill

21. I teach students how to behave safely and responsibly online

A	This is not possible in my subject or work environment
B	I inform you that you should be careful when transmitting personal information online
C	I explain the basic rules for acting safely and responsibly in online environments
D	We discuss and agree on rules of conduct online
E	We systematically develop social rules for students in the different digital environments we use

22. I encourage students to use digital technologies creatively to solve specific problems. For example, overcoming obstacles or emerging challenges in your learning process

A	This is not possible with my students due to the work environment
B	I rarely have the opportunity to encourage students' digital problem solving
C	Occasionally, whenever an opportunity arises
D	We often experiment with technological solutions to specific problems
E	I systematically integrate tasks for creative digital problem solving

Section E: This section is about yours feeling of your job. Please read statements and rate accordingly.

**Statements**	Strongly Disagree	Disagree	Neutral	Agree	Strongly Agree
**1.** I feel emotionally drained from my work.					
**2.** I feel used up at the end of the workday.					
**3.** I feel fatigued when I get up in the morning and have to face another day on the job.					
**4.** Working with people all day is really a strain for me.					
**5.** I feel burned out from my work.					
**6.** I feel frustrated by my job.					
**7.** I feel I'm working too hard on my job.					
**8.** Working with people directly puts too much stress on me.					
**9.** I feel like I'm at the end of my rope.					
**10.** I feel I treat some students as if they were impersonal objects.					
**11.** I've become more callous toward people since I took this job.					
**12.** I worry that this job is hardening me emotionally.					

**13.** I don't care what happens to some students.					
**14.** I feel students blame me for some of their problems.					
**15.** I can easily understand how my students feel about things.					
**16.** I deal very effectively with the problems of my students.					
**17.** I feel I'm positively influencing other people's lives through my work.					
**18.** I feel very energetic.					
**19.** I can easily create a relaxed atmosphere with my students.					
**20.** I feel exhilarated after working closely with my students.					
**21.** I have accomplished many worthwhile things in this job.					
**22.** In my work, I deal with emotional problems very calmly.					

Thanks for your participation.
